# Maternal Cardiovascular Phenotype in Fetal Growth Restriction with or Without Pre-Eclampsia: Insights from Echocardiography and Clinical Implications

**DOI:** 10.3390/diagnostics16152414

**Published:** 2026-07-31

**Authors:** Dorina-Adelina Minciuna, Mihai Stefan Cristian Haba, Raluca-Maria Haba, Cosmin-Daniel Minciuna, Alexandru Carauleanu, Ioana-Sadiye Scripcariu, Cristina David, Daniela-Cristina Dimitriu, Demetra-Gabriela Socolov

**Affiliations:** 1 Grigore T. Popa University of Medicine and Pharmacy, 700115 Iasi, Romania; dorina-adelina.minciuna@umfiasi.ro (D.-A.M.); ralucavreme@yahoo.com (R.-M.H.); cosmin.minciuna@umfiasi.ro (C.-D.M.); acarauleanu@yahoo.com (A.C.); isscripcariu@gmail.com (I.-S.S.); crisdavid2003@yahoo.com (C.D.); daniela.dimitriu@umfiasi.ro (D.-C.D.); 2 “Saint Spiridon” County Clinical Emergency Hospital, 700111 Iasi, Romania; 3 Regional Institute of Oncology, 700483 Iasi, Romania; 4 Clinical Hospital of Obstetrics and Gynecology “Cuza Voda”, 700038 Iasi, Romania

**Keywords:** fetal growth restriction, maternal hemodynamics, echocardiography, cardiac output, systemic vascular resistance, cardiovascular phenotype, preeclampsia, diastolic dysfunction, pregnancy complications

## Abstract

Fetal growth restriction (FGR) has traditionally been attributed to placental insufficiency. However, accumulating evidence suggests that maternal cardiovascular maladaptation contributes to disease expression, clinical heterogeneity, and long-term maternal cardiovascular risk. To review maternal echocardiographic findings in pregnancies complicated by FGR, with or without preeclampsia and to explore the role of cardiovascular phenotyping in risk stratification and clinical management. Narrative review of the current literature, including observational studies, systematic reviews, meta-analyses, and expert consensus statements on maternal hemodynamics and echocardiographic assessment in FGR. FGR is associated with a maladaptive cardiovascular profile characterized by reduced cardiac output, increased systemic vascular resistance and adverse cardiac remodeling. Echocardiography frequently identifies subclinical myocardial dysfunction, including impaired diastolic function and reduced global longitudinal strain despite preserved ejection fraction. Distinct maternal cardiovascular phenotypes emerge across the FGR spectrum, ranging from high-risk hypodynamic, resistance-dominant phenotypes to intermediate and low-risk phenotypes with preserved hemodynamic adaptation. Integration of maternal cardiovascular assessment with fetal Doppler findings may improve risk stratification and support phenotype-guided surveillance and therapeutic decision-making. Persistent postpartum cardiovascular abnormalities further support the concept of pregnancy as a cardiovascular stress test and identify women at increased long-term cardiovascular risk. FGR should be viewed as the clinical manifestation of a complex maternal–placental–cardiovascular interaction rather than an isolated placental disorder. Maternal cardiovascular phenotyping has the potential to refine risk stratification, support hemodynamic-guided management, and identify women who may benefit from structured long-term cardiovascular surveillance.

## 1. Introduction

Fetal growth restriction (FGR) remains one of the most significant complications of pregnancy, affecting approximately 4–8% of gestations and representing a leading cause of perinatal morbidity and mortality worldwide [1,2,3,4]. According to the Delphi consensus definition, subsequently endorsed by international societies including FIGO and ISUOG, FGR is diagnosed antenatally when the estimated fetal weight (EFW) or abdominal circumference (AC) is below the 3rd percentile, or when fetuses with an EFW or AC below the 10th percentile meet additional Doppler criteria according to gestational age [5,6,7]. Despite significant advancements in prenatal surveillance, the antenatal detection of FGR remains suboptimal. Current evidence suggests that approximately 70–75% of affected pregnancies are not recognized before delivery and are identified only retrospectively after birth, reflecting missed antenatal diagnosis [5,8].

Traditionally, FGR has been regarded as a disorder predominantly driven by placental dysfunction. Beyond diagnosis, Doppler assessment is essential for evaluating placental insufficiency, stratifying disease severity, guiding fetal surveillance and the timing of delivery. However, growing evidence indicates that this placenta-centered diagnostic paradigm does not fully capture the complex interaction between maternal, placental and fetal factors underlying FGR [5,6,7,9,10,11]. Instead, FGR is increasingly viewed within a broader maternal–placental–cardiovascular framework, in which maternal cardiovascular maladaptation is closely associated with disease severity and is reflected by a characteristic phenotype of reduced cardiac output (CO) and increased systemic vascular resistance (SVR) [12,13,14,15,16,17,18].

This shift is particularly relevant considering the well-established association between FGR and hypertensive disorders of pregnancy, especially preeclampsia. Both conditions share common pathophysiological pathways, including impaired placentation, endothelial dysfunction, and maternal cardiovascular maladaptation [19]. Importantly, these cardiovascular alterations may precede the clinical diagnosis and can persist postpartum, suggesting that FGR may represent an early marker of maternal cardiovascular vulnerability [20,21].

In this context, maternal echocardiography has emerged as a key tool for the non-invasive assessment of cardiovascular function during pregnancy. Beyond conventional parameters such as cardiac output, advanced techniques including tissue Doppler imaging and myocardial deformation analysis (speckle-tracking-derived strain) allow the detection of early, subclinical myocardial impairment, refining risk stratification in conditions such as fetal growth restriction and preeclampsia [19,22].

The aim of this review is to synthesize a comprehensive overview of maternal echocardiographic assessment in pregnancies complicated by FGR, with or without preeclampsia, focusing on the identification of distinct cardiovascular phenotypes and their clinical implications for diagnosis, risk stratification, and personalized physiology-based management through systematically incorporating maternal hemodynamic profiling into diagnostic management algorithms [17,23].

## 2. Literature Search Strategy

A structured literature search was conducted using three electronic databases: PubMed, Scopus, and Web of Science. The search strategy combined Medical Subject Headings (MeSH) and free-text terms, including: “fetal growth restriction”, “intrauterine growth restriction”, “maternal hemodynamics”, “maternal cardiovascular adaptation”, “echocardiography”, “cardiac output”, “stroke volume”, “systemic vascular resistance”, “global longitudinal strain”, “speckle-tracking echocardiography”, “cardiac remodeling”, and “preeclampsia”. Reference lists of relevant articles were also manually screened to identify additional eligible publications. The search identified 841 records across PubMed (*n* = 241), Scopus (*n* = 330), and Web of Science (*n* = 270). Following duplicate removal and title/abstract screening for relevance, 172 potentially relevant articles were retained for full-text assessment. The most relevant studies were subsequently selected for inclusion in the narrative synthesis.

The electronic literature search primarily covered publications published in English between January 2016 and May 2026. Earlier landmark studies that established the current concepts of maternal cardiovascular adaptation, ventricular remodeling and hemodynamic assessment in FGR and preeclampsia were also included when considered essential for understanding the evolution of the field.

The literature search and study selection were performed independently by two authors (D.-A.M. and M.S.C.H.), with disagreements resolved by discussion and, when necessary, consultation with the senior author (D.-G.S.).

The review preferentially included original observational (prospective and retrospective) and interventional studies, systematic reviews, meta-analyses, clinical practice guidelines and expert consensus statements. Studies were eligible if they evaluated pregnancies complicated by FGR, with or without preeclampsia and investigated maternal cardiovascular structure and function using echocardiography or validated non-invasive hemodynamic monitoring techniques. Emphasis was placed on studies reporting quantitative maternal cardiovascular parameters, such as cardiac output, stroke volume, systemic vascular resistance, ventricular geometry and remodeling, systolic and diastolic function and myocardial deformation indices (particularly global longitudinal strain).

Throughout this review, the term FGR refers to the antenatal Delphi consensus definition unless otherwise specified.

Studies were excluded if they consisted solely of case reports, conference abstracts without full-text publication, editorials, commentaries, letters without original data, or animal and experimental studies. Studies focusing exclusively on fetal or placental assessment without evaluation of maternal cardiovascular function were also excluded, as were duplicate publications and studies with insufficient methodological description or without quantitative maternal cardiovascular outcomes. Studies involving multiple pregnancies or major fetal structural or chromosomal anomalies were excluded unless separate analyses for pregnancies complicated by FGR were available.

The selected literature was synthesized narratively, focusing on the pathophysiological links between placental dysfunction and maternal cardiovascular maladaptation, hemodynamic phenotypes, differences between isolated FGR and FGR associated with preeclampsia, and their clinical implications.

## 3. From Placental Disease to Maternal Cardiovascular Syndrome—Pathophysiology “Beyond the Placenta”

Historically, FGR has been defined as a clinical consequence of placental insufficiency, resulting from defective trophoblastic invasion and suboptimal spiral arteries remodeling [16,24,25]. However, this placenta-centered paradigm does not fully explain the heterogeneity of FGR or its close association with maternal cardiovascular dysfunction [19,26].

Increasing evidence supports a broader maternal–placental–cardiovascular model, in which placental dysfunction and maternal cardiovascular maladaptation appear to represent interconnected processes rather than isolated events [12,17,19]. Normal placentation is critically dependent on adequate maternal perfusion and the physiological transformation of spiral arteries into low-resistance vessels ensures a high-flow uteroplacental circulation. However, this process is highly sensitive to maternal hemodynamic conditions. Suboptimal cardiovascular adaptation characterized by insufficient plasma volume expansion, impaired vascular adaptation and limited cardiac reserve may compromise uterine perfusion and contribute to abnormal placental development [10,12,24,27,28].

Importantly, prospective longitudinal cohorts have demonstrated that women who subsequently develop FGR often exhibit subclinical cardiovascular abnormalities, including reduced cardiac output and increased vascular resistance, from early pregnancy, suggesting that maternal cardiovascular dysfunction may precede the clinical manifestation of placental disease [13,29,30,31].

Endothelial dysfunction appears to represent a key mechanistic link between maternal cardiovascular impairment and placental disease. An imbalance between anti-angiogenic factors, such as soluble fms-like tyrosine kinase-1 (sFlt-1), and pro-angiogenic mediators, including placental growth factor (PlGF), leads to systemic vasoconstriction, increased afterload, and reduced vascular compliance. These changes impair not only maternal systemic circulation but also uteroplacental perfusion, establishing a self-perpetuating cycle of placental hypoxia and fetal compromise [19,32,33,34,35].

The overlap between FGR and hypertensive disorders of pregnancy further supports this concept characterized by endothelial dysfunction, increased vascular resistance, and adverse cardiac remodeling, with preeclampsia likely representing a more severe systemic manifestation of the same underlying process [19,26,36,37]. Taken together, these findings support an evolving paradigm in which FGR is increasingly viewed as a placental disorder closely linked to maternal cardiovascular maladaptation. This perspective emphasizes the bidirectional interaction between maternal hemodynamics and placental function and highlights the central role of maternal cardiovascular adaptation in determining pregnancy outcome.

**Figure 1:**
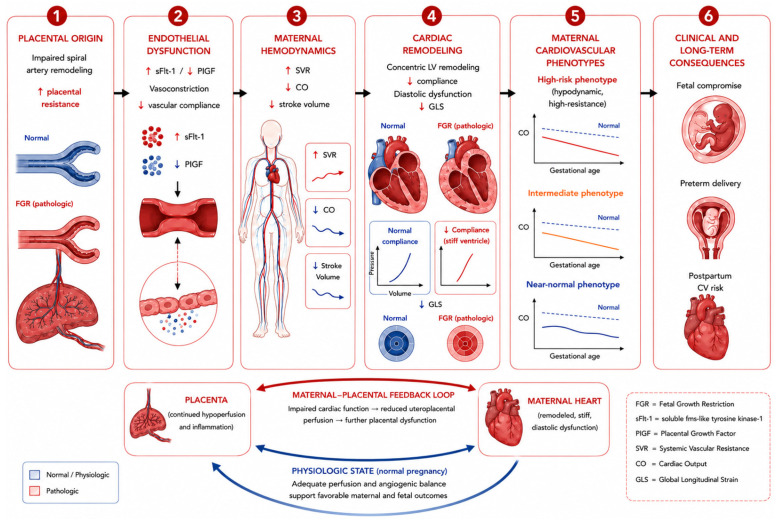
Proposed maternal–placental–cardiovascular model of fetal growth restriction. Impaired spiral artery remodeling and placental dysfunction promote endothelial imbalance, characterized by increased sFlt-1 and reduced PlGF levels, leading to systemic vasoconstriction, elevated vascular resistance, and reduced cardiac output. Progressive maternal hemodynamic maladaptation contributes to adverse cardiac remodeling, impaired myocardial function, and the emergence of distinct cardiovascular phenotypes. A bidirectional feedback loop between placental hypoperfusion and maternal cardiovascular dysfunction may further exacerbate disease severity, ultimately influencing fetal outcomes and long-term maternal cardiovascular risk. Arrows indicate the proposed sequence of pathophysiological events, while upward (↑) and downward (↓) arrows denote increases and decreases, respectively, in the corresponding parameters. Conceptual illustration created by the authors with the assistance of OpenAI ChatGPT (GPT-5.5; Open AI, San Francisco, CA, USA) for graphical design.

Figure 1 summarizes the proposed pathophysiological continuum linking placental dysfunction, endothelial impairment, maternal cardiovascular maladaptation, and the development of distinct maternal cardiovascular phenotypes in FGR.

## 4. Maternal Cardiovascular Adaptation vs. Maladaptation in FGR

Pregnancy is characterized by a series of profound and tightly regulated cardiovascular adaptations that begin in early gestation and progressively increase to meet the growing metabolic demands of the fetus while ensuring adequate uteroplacental perfusion. These hemodynamic changes result from the intricate interplay between vascular remodeling, adaptive cardiac mechanics, and complex neurohormonal signaling [38,39].

Physiological adaptation in pregnancy consists of a high-output, low-resistance circulation that is essential for maintaining adequate uteroplacental blood flow and supporting fetal development. Failure to achieve or sustain this hemodynamic profile may compromise placental perfusion and has been increasingly implicated in the pathogenesis of fetal growth restriction, highlighting the central role of maternal cardiovascular adaptation in determining pregnancy outcome [22,23,29,38,40,41,42,43,44,45].

In pregnancies complicated by FGR, this adaptive process appears to be altered. Instead of the expected hyperdynamic circulation, women with FGR frequently exhibit a maladaptive hemodynamic profile characterized by reduced cardiac output and increased systemic vascular resistance [12,16,26,27,30,46]. This pattern reflects an inability of the maternal cardiovascular system to appropriately respond to the hemodynamic demands of pregnancy and provides the substrate for the echocardiographic abnormalities observed in FGR.

## 5. Echocardiographic Assessment: Parameters and Interpretation

Maternal echocardiography is a valuable non-invasive method for evaluation of cardiovascular adaptation during pregnancy. In FGR, echocardiographic assessment allows the identification of subclinical cardiovascular abnormalities that are not detected by conventional clinical assessment [12,23,28,29,47,48].

Most evidence is derived from observational studies performed after the diagnosis of FGR or in women at increased risk of placental disease, rather than from cohorts with pre-pregnancy cardiovascular assessment. Consequently, it remains uncertain whether the observed abnormalities reflect pre-existing maternal susceptibility, pregnancy-induced maladaptation, or both. Although diastolic dysfunction and impaired global longitudinal strain (GLS) are consistently reported, the prevalence of right ventricular dysfunction and pulmonary hemodynamic abnormalities remains uncertain because of substantial heterogeneity in study populations, disease severity, echocardiographic protocols, and hemodynamic assessment techniques [28,47,49,50].

Furthermore, while several longitudinal studies have shown that maternal cardiovascular dysfunction may precede the clinical manifestation of placental insufficiency, findings are less consistent in late-onset FGR, where cardiac output may remain relatively preserved in some patients [17,28,29,30,51].

### 5.1. Core Hemodynamic Parameters

The assessment of maternal cardiovascular status is primarily based on the evaluation of cardiac output, stroke volume, and systemic vascular resistance, which together define the global hemodynamic profile [23,30,52,53,54].

In normal pregnancy, echocardiography demonstrates a hyperdynamic circulation characterized by increased CO and reduced SVR. In contrast, pregnancies complicated by FGR consistently show significantly lower CO and higher SVR, reflecting a maladaptive hemodynamic state, with more pronounced alterations in early-onset disease [12,15,26,27,28,46,55].

### 5.2. Left Ventricular (LV) Structure and Remodeling

Beyond global hemodynamics, echocardiography allows detailed evaluation of cardiac structure. In FGR, the LV frequently exhibits concentric remodeling or hypertrophy, characterized by an increased left ventricular mass index and relative wall thickness. This pattern contrasts with the eccentric remodeling observed in normal pregnancy and reflects adaptation to increased afterload.

Concentric geometry is associated with impaired ventricular compliance and reduced cardiovascular reserve. It is more pronounced in early-onset FGR and pregnancies complicated by preeclampsia and correlates with disease severity, representing an important marker of maternal cardiovascular maladaptation [12,38,39].

### 5.3. Systolic Function: Beyond Ejection Fraction

In addition to global hemodynamic alterations, myocardial function is frequently affected in pregnancies complicated by FGR [15]. Although conventional measures of systolic function, such as preserved left ventricular ejection fraction (LVEF), are generally preserved, advanced echocardiographic techniques have revealed evidence of subclinical myocardial dysfunction [12,55,56]. Global longitudinal strain, assessed by speckle-tracking echocardiography, is a sensitive marker of myocardial deformation that can detect early systolic impairment before changes in LVEF become apparent [12,57,58,59,60].

Women with FGR consistently demonstrate less negative GLS values, reflecting impaired longitudinal myocardial fiber contraction despite preserved global systolic performance. These findings indicate that myocardial dysfunction in FGR extends beyond conventional measures of systolic function and may contribute to disease severity and cardiovascular risk stratification [12,19,49,58,59,61].

### 5.4. Diastolic Function: An Early Marker of Maladaptation

Diastolic dysfunction is increasingly recognized as one of the earliest manifestations of maternal cardiovascular maladaptation in pregnancies complicated by FGR. Reduced early diastolic myocardial velocity (e′) and increased E/e′ ratio reflect impaired ventricular relaxation and elevated filling pressures, reflecting increased myocardial stiffness secondary to chronic afterload elevation [12,13,22,23,55,60,62,63,64]. These abnormalities may be accompanied by an increased left atrial volume index, a marker of chronically elevated filling pressures and adverse cardiac remodeling.

Diastolic dysfunction, often accompanied by impaired global longitudinal strain, frequently precedes overt systolic dysfunction, while left ventricular ejection fraction generally remains preserved until more advanced stages of maternal cardiovascular maladaptation. Evidence from echocardiographic studies demonstrates that the severity of diastolic dysfunction correlates with disease severity and is particularly pronounced in early-onset FGR and pregnancies complicated by preeclampsia [40,65,66,67,68,69]. These findings identify diastolic dysfunction as a key component of the maternal cardiovascular phenotype associated with placental disease.

### 5.5. Right Ventricular Function and Pulmonary Circulation

Evaluation of the right heart and pulmonary circulation provides critical insights into the systemic nature of maternal cardiovascular maladaptation in FGR. Emerging evidence suggests that women with FGR may exhibit subtle impairment of right ventricular (RV) function, reflected by reduced tricuspid annular plane systolic excursion (TAPSE), impaired RV longitudinal mechanics, and, in severe cases, mildly elevated pulmonary arterial pressures [23,28,31,70].

These findings indicate that the hemodynamics of FGR are not restricted to the LV but rather reflect a global, biventricular cardiovascular involvement. Identifying RV dysfunction serves as a marker for advanced disease and higher systemic hemodynamic strain. While this remains an emerging area of research, recent observational cohorts suggest that biventricular assessment significantly enhances the characterization of the maternal cardiovascular phenotype in FGR [29,52,71].

The principal cardiovascular differences between normal pregnancy, late-onset FGR, and early-onset FGR are summarized in Table 1.

## 6. Maternal Cardiovascular Phenotypes in FGR with or Without Preeclampsia

The integration of hemodynamic, structural, and functional cardiovascular parameters has led to the recognition of distinct maternal cardiovascular phenotypes in pregnancies complicated by FGR. This phenotype-based approach extends beyond traditional size-based classifications by providing a more comprehensive characterization of maternal cardiovascular adaptation, placental disease severity and clinical risk [12,13,52,72].

Hemodynamic studies suggest that maternal cardiovascular adaptation may broadly follow either a resistance-dominant or a volume-dominant pattern. In FGR, the resistance-dominant profile, characterized by reduced cardiac output, elevated systemic vascular resistance, and impaired uteroplacental perfusion is the most clinically relevant and is frequently associated with placental insufficiency, cardiac remodeling, and adverse perinatal outcomes [23,26,31]. In contrast, volume-dominant patterns are generally associated with preserved placental perfusion and normal fetal growth [23].

The severity of these alterations appears to correlate with the clinical presentation of FGR. Early-onset FGR is typically associated with more pronounced cardiovascular impairment, including marked reductions in cardiac output and significantly elevated vascular resistance, reflecting a severe hypodynamic state. In contrast, late-onset FGR often presents with subtler abnormalities, frequently dominated by diastolic dysfunction with relatively preserved cardiac output, suggesting a milder or later manifestation of the same pathophysiological continuum [23,26,28,29,52,60,73].

### 6.1. High-Risk (Hypodynamic) Phenotype

The high-risk hypodynamic phenotype represents the most severe form of maternal cardiovascular maladaptation in pregnancies complicated by FGR. It is characterized by the most severe hemodynamic impairment, concentric remodeling and marked myocardial dysfunction [12,23,26,74,75,76].

This phenotype is most frequently observed in early-onset FGR and in pregnancies complicated by preeclampsia, where abnormal E/e′ values have been associated with increased ventricular stiffness, adverse cardiac remodeling, and early myocardial dysfunction [55,60,77]. The combination of reduced forward flow and increased afterload limits the maternal capacity to augment uteroplacental perfusion, thereby contributing to severe placental insufficiency, abnormal uterine artery Doppler findings, and progressive fetal compromise [17,28,29,30,52,78,79].

Studies by Katherine Melchiorre et al. consistently demonstrate that this cardiovascular profile is associated with the greatest degree of hemodynamic impairment and the highest risk of adverse perinatal outcomes, including progressive fetal compromise and medically indicated preterm delivery [12,27]. Consequently, the hypodynamic phenotype may be regarded as the prototypical high-risk cardiovascular phenotype in FGR, highlighting the potential role of cardiovascular phenotyping in risk stratification, clinical decision making and postpartum cardiovascular surveillance.

### 6.2. Intermediate Phenotype

The intermediate phenotype is most commonly observed in late-onset FGR and is characterized by relatively preserved or mildly reduced cardiac output, moderate increase in SVR, evidence of diastolic dysfunction, and minimal or absent structural remodeling [13,17,23,28,29,30]. This profile suggests a partial failure of maternal cardiovascular adaptation, where compensatory mechanisms remain operational but are constrained by a progressively diminishing functional reserve. In this context, diastolic dysfunction appears as the primary hemodynamic dysfunction, manifesting as increased ventricular stiffness and impaired myocardial relaxation despite the maintenance of global systolic performance.

Clinically, this phenotype is often associated with less severe placental disease, milder Doppler abnormalities and a more gradual progression of fetal compromise compared to high-risk hypodynamic profiles. Nevertheless, the presence of reduced cardiovascular reserve suggests an increased susceptibility to adverse maternal and perinatal outcomes, particularly as placental dysfunction progresses.

### 6.3. Low-Risk (Near-Normal) Phenotype

The low-risk or near-normal phenotype is most frequently observed in constitutionally small fetuses and in selected cases of mild late-onset FGR. It is characterized by preserved cardiac output, normal or minimally elevated systemic vascular resistance, and largely preserved systolic and diastolic function, with little or no evidence of adverse cardiac remodeling [74,80]. Echocardiographic assessment typically demonstrates preserved global longitudinal strain, normal diastolic indices (normal e′ velocity and E/e′ ratio), and the absence of significant concentric remodeling. These findings contrast with the intermediate phenotype, in which subtle diastolic abnormalities and mild increases in vascular resistance are already present despite relatively preserved cardiac output. In these cases, the absence of significant maternal cardiovascular maladaptation suggests that fetal smallness may not primarily result from severe placental insufficiency but rather reflects constitutional, genetic or less pronounced placental factors [17]. Clinically, this phenotype is associated with a favorable cardiovascular profile, near-normal Doppler findings, and a lower risk of adverse perinatal outcomes. Recognition of this phenotype is particularly important, as it may help distinguish constitutionally small fetuses from pathological FGR, thereby avoiding unnecessary intervention and supporting a more conservative management strategy. Based on these observations, pregnancies complicated by FGR can be broadly categorized into three maternal cardiovascular phenotypes: a high-risk hypodynamic phenotype, an intermediate phenotype, and a low-risk near-normal phenotype (Table 2). Beyond their descriptive value, these cardiovascular profiles provide clinically relevant information regarding disease severity, progression, prognosis, and potential therapeutic response, thereby supporting a more individualized approach to risk stratification and management [23,26,46,72,81].

Table 2 classification is based on the degree of maternal cardiovascular maladaptation and integrates hemodynamic, echocardiographic, placental, and clinical characteristics. The model highlights the continuum from preserved cardiovascular adaptation to severe hypodynamic maladaptation and its potential implications for risk stratification, management, and long-term cardiovascular surveillance.

Importantly, these maternal cardiovascular phenotypes should not be conceptualized as static categories but rather as a dynamic continuum of cardiovascular adaptation. As placental dysfunction progresses, patients may transition from a near-normal or intermediate phenotype toward a more severe hypodynamic profile [82]. This concept is supported by longitudinal studies demonstrating gradual deterioration in maternal hemodynamics in pregnancies complicated by FGR, with increasing vascular resistance and decreasing cardiac output over time [29]. This continuum framework provides the rationale for integrating cardiovascular phenotyping into risk stratification, clinical decision-making, and postpartum cardiovascular surveillance.

## 7. Clinical Implications: Diagnosis, Risk Stratification, Therapy

Recognizing FGR as a manifestation of maternal cardiovascular maladaptation has important implications for clinical practice. Beyond a placenta-centered approach, maternal cardiovascular assessment provides additional information regarding disease severity, risk stratification, therapeutic decision-making (based on CO-SVR profile), and postpartum cardiovascular surveillance [17,26,27]. However, current evidence supports a targeted rather than routine approach because of limited availability of expertise, equipment and standardized protocols.

### 7.1. Diagnostic Implications: Beyond Fetal and Placental Assessment

Current diagnostic algorithms for FGR rely predominantly on fetal biometry and Doppler assessment of the uteroplacental and fetoplacental circulation. Although these tools remain fundamental, they do not capture the maternal cardiovascular contribution to disease pathophysiology [5,6].

Maternal echocardiography can identify subclinical cardiovascular dysfunction before overt fetal compromise becomes apparent, even in normotensive pregnancies. This additional information could improve phenotyping and help distinguish pathological FGR from constitutionally small fetuses [27,29,30]. Furthermore, combining maternal cardiovascular findings with Doppler abnormalities may improve assessment of disease severity and identify women at the highest risk of adverse outcomes [46,83].

### 7.2. Risk Stratification and Monitoring

Maternal cardiovascular assessment may complement conventional fetal and placental evaluation by refining risk stratification in pregnancies complicated by FGR. Echocardiographic findings may support individualized surveillance strategies, guide referral to tertiary care centers, and contribute to decisions regarding monitoring intensity and timing of delivery [23,84]. Based on the available evidence, maternal echocardiography may be particularly valuable in women with early-onset FGR, FGR associated with preeclampsia, severe placental insufficiency, previous placental disease or significant maternal cardiovascular risk factors. In these patients, cardiovascular assessment may support multidisciplinary management, individualized surveillance, and delivery planning.

### 7.3. Therapeutic Perspectives: Toward Hemodynamic-Guided Treatment

Cardiovascular phenotyping provides the foundation for hemodynamic-guided therapy, an emerging strategy in which treatment selection is based on the underlying maternal cardiovascular profile rather than blood pressure values alone [23,26,29,31,85,86,87].

In patients with a low-output, high-resistance phenotype, vasodilatory agents such as calcium channel blockers (e.g., nifedipine) may be beneficial by reducing systemic vascular resistance and improving cardiac output. Although emerging hemodynamic studies suggest that beta-blockers such as labetalol may be less favorable in women with hypodynamic phenotypes, this concept remains investigational and has not yet been incorporated into current international clinical guidelines. Therefore, antihypertensive therapy should continue to follow established guideline recommendations while future prospective studies determine whether phenotype-guided treatment strategies improve maternal and perinatal outcomes [26,85,86,87,88,89].

Importantly, the clinical value of cardiovascular phenotyping extends beyond pregnancy. Women exhibiting adverse cardiovascular profiles, particularly hypodynamic, resistance-dominant phenotypes, appear more likely to develop persistent postpartum hemodynamic abnormalities and may represent a subgroup at increased risk of future cardiovascular disease. Although it remains uncertain whether cardiovascular dysfunction precedes placental disease or develops in parallel with it, the identification of these phenotypes provides a unique opportunity for long-term cardiovascular surveillance and preventive intervention. In this context, pregnancy may serve as an early cardiovascular stress test, identifying women who may benefit from structured long-term cardiovascular follow-up.

### 7.4. Preventive Strategies and Lifestyle Interventions

Although evidence supporting phenotype-guided pharmacological therapy remains limited, preventive strategies remain an essential component of cardiovascular care in women at risk of placental disease.

Lifestyle optimization, including adherence to a healthy dietary pattern, regular moderate physical activity, weight optimization before pregnancy and smoking cessation may improve endothelial function and maternal cardiovascular adaptation. Low-dose aspirin remains the only pharmacological intervention currently recommended by major international guidelines for women at high risk of preeclampsia, where it reduces the incidence of preeclampsia and related placental complications when initiated before 16 weeks of gestation. Although statins have shown promising biological effects in experimental studies, current evidence remains insufficient to recommend their routine use for prevention or treatment of FGR outside clinical trials. Following delivery, lifestyle modification and cardiovascular risk assessment should form an integral part of long-term follow-up, particularly in women with high-risk cardiovascular phenotypes [90].

## 8. Postpartum Cardiovascular Risk and the “Fourth Trimester”

The concept of the “fourth trimester” recognizes this period as critical for understanding the long-term cardiovascular consequences of pregnancies complicated by fetal growth restriction. Increasing evidence suggests that maternal cardiovascular maladaptation may persist beyond delivery, particularly in women with high-risk hemodynamic phenotypes [23]. Several studies have demonstrated that women with FGR exhibit persistent hemodynamic abnormalities after delivery, including reduced cardiac output and elevated systemic vascular resistance, even at 3–6 months postpartum [28,89,91].

This observation supports the concept of pregnancy as a cardiovascular stress test, whereby pregnancy may unmask latent cardiovascular susceptibility. Women with a history of FGR have an increased long-term risk of chronic hypertension, ischemic heart disease, heart failure, and stroke, especially in cases of early-onset disease or coexisting preeclampsia [23,48,65,92,93,94]. Within this framework, FGR may be regarded not only as a marker of placental dysfunction but also as an early indicator of future maternal cardiovascular vulnerability.

Beyond maternal cardiovascular health, the concept of the fourth trimester may also be viewed within the framework of the maternal–fetal dyad. The maternal hemodynamic abnormalities that characterize pregnancies complicated by FGR, including reduced cardiac output, increased systemic vascular resistance, and endothelial dysfunction, may influence fetal cardiovascular development through mechanisms of developmental programming. Consequently, offspring exposed to these adverse intrauterine conditions may exhibit persistent alterations in cardiovascular structure and function, predisposing them to hypertension, metabolic disorders, and cardiovascular disease later in life. This transgenerational perspective further supports the concept that maternal cardiovascular maladaptation has consequences extending well beyond pregnancy and emphasizes the importance of long-term follow-up for both mother and child [90].

Cardiovascular phenotyping may represent a valuable approach for identifying those women at greatest long-term risk. In particular, patients exhibiting a hypodynamic, high-resistance profile appear more likely to demonstrate persistent postpartum abnormalities and may benefit from structured cardiovascular surveillance during the fourth trimester and beyond [17,19,48]. Maternal echocardiography may play a pivotal role in the postpartum evaluation of women with FGR. Assessment of cardiac output, diastolic function, and left ventricular geometry can identify persistent cardiovascular impairment and facilitate risk stratification after delivery [23,28,58,89].

Although routine postpartum cardiovascular screening has not yet been incorporated into clinical guidelines, current evidence supports the development of integrated cardio-obstetric follow-up pathways for women with high-risk FGR phenotypes [17,23,28,85].

Future postpartum care pathways may therefore evolve from maternal cardiovascular surveillance alone toward integrated maternal-offspring cardiovascular prevention strategies [90].

## 9. Future Perspectives: From Cardiovascular Phenotyping to Precision Cardio-Obstetrics

### 9.1. Standardization of Maternal Cardiovascular Phenotyping

Despite increasing evidence supporting maternal cardiovascular assessment in FGR, its translation into routine clinical practice remains limited by the lack of standardized imaging protocols, validated hemodynamic reference ranges, and universally accepted definitions of maternal cardiovascular phenotypes [19,48,91]. Future multicenter prospective studies should validate reproducible cardiovascular phenotypes, establish clinically relevant thresholds and support incorporation of cardiovascular phenotyping into evidence-based clinical guidelines [95].

### 9.2. Advanced Cardiovascular Assessment and Artificial Intelligence

Emerging technologies including speckle-tracking imaging, myocardial strain analysis, and myocardial work analysis may detect subclinical myocardial dysfunction [12,19,48]. and facilitate integrated assessment combining maternal echocardiography with fetal Doppler velocimetry, circulating biomarkers such as angiogenic biomarkers (sFlt-1, PlGF) and myocardial stress biomarker (NT-proBNP), placental imaging and clinical characteristics [52,95].

Artificial intelligence and machine-learning approaches may further enhance multimodal cardiovascular assessment by integrating echocardiographic, Doppler, biomarker, and clinical data into comprehensive prediction models. Although these technologies are promising, their clinical application in pregnancies complicated by FGR remains preliminary and requires prospective validation before routine implementation.

### 9.3. Toward Precision Cardio-Obstetrics

The emphasis should now shift from descriptive cardiovascular assessment to the identification of clinically actionable maternal cardiovascular phenotypes that can guide individualized surveillance, hemodynamic-guided therapy, delivery planning and structured postpartum cardiovascular follow-up [19,48,95]. Women with hypodynamic, high-resistance profiles may benefit from intensified maternal–fetal surveillance, phenotype-guided antihypertensive therapy, individualized delivery planning, and structured postpartum cardiovascular follow-up, whereas those with preserved cardiovascular adaptation may require less intensive surveillance.

Future clinical algorithms will likely integrate maternal echocardiographic parameters, fetal Doppler findings, circulating biomarkers, placental imaging and, potentially, artificial intelligence-assisted phenotyping into a unified precision cardio-obstetric model, supporting personalized surveillance, hemodynamic-guided therapy and lifelong cardiovascular prevention [52,95].

Such an approach may also support primordial cardiovascular prevention within the maternal–fetal dyad by identifying women and offspring at increased cardiovascular risk early in life, thereby creating opportunities for preventive interventions that extend beyond pregnancy [90].

Future research should focus on validating clinically actionable maternal cardiovascular phenotypes, establishing standardized multimodal hemodynamic assessment protocols, and determining whether phenotype-guided surveillance and therapeutic strategies can improve perinatal outcomes while reducing long-term maternal cardiovascular risk.

## 10. Strengths and Limitations

This review has several strengths. It integrates current evidence on maternal cardiovascular adaptation and maladaptation in fetal growth restriction, highlighting the emerging concept of maternal cardiovascular phenotyping and its potential clinical implications. It also incorporates recent international guidelines and expert consensus statements, including the latest ISUOG recommendations, while placing echocardiographic findings within a clinically relevant cardio-obstetric framework.

Several limitations should also be acknowledged. As a narrative review, this manuscript provides a qualitative synthesis of the available evidence and did not include a formal quantitative risk-of-bias assessment. Furthermore, much of the available evidence is derived from heterogeneous observational studies that differ in study populations, echocardiographic methodologies, and definitions of maternal cardiovascular phenotypes, limiting direct comparison across studies. Finally, although accumulating evidence supports the potential clinical value of maternal cardiovascular assessment in FGR, prospective multicenter studies are still needed before phenotype-guided management can be broadly implemented.

## 11. Conclusions

Fetal growth restriction should no longer be viewed solely as a placental disorder, but rather as the clinical manifestation of a complex maternal–placental–cardiovascular interaction. Growing evidence indicates that maternal cardiovascular maladaptation is closely associated with disease severity and contributes to the marked heterogeneity observed among pregnancies complicated by FGR.

Perhaps the most important advance in recent years has been the recognition of distinct maternal cardiovascular phenotypes, ranging from preserved cardiovascular adaptation to intermediate and high-risk hypodynamic, resistance-dominant profiles. These phenotypes appear to reflect different degrees of placental dysfunction and may represent the mechanistic link between placental disease, adverse perinatal outcomes, persistent postpartum cardiovascular abnormalities, and future maternal cardiovascular risk.

Maternal echocardiography offers a practical, non-invasive tool for identifying these phenotypes and may improve diagnosis, risk stratification, surveillance strategies, and therapeutic decision-making through mechanism-based hemodynamic assessment.

This integrated maternal cardiovascular perspective also reinforces the concept of the maternal–fetal dyad, recognizing that maternal cardiovascular maladaptation may influence not only pregnancy outcomes but also the long-term cardiovascular health of the offspring.

Ultimately, integrating maternal cardiovascular phenotyping into FGR evaluation has the potential to redefine the clinical management of placental disease, bridging obstetric care and cardiovascular medicine while transforming pregnancy into an opportunity for lifelong cardiovascular prevention.

## Figures and Tables

**Table 1:** Maternal cardiovascular adaptation in normal pregnancy, late-onset and early-onset fetal growth restriction.

	Parameter	Normal Pregnancy	Late FGR (≥32 Weeks)	Early FGR (<32 Weeks)
**Core hemodynamic parameters**	Cardiac output	↑↑	↓	↓↓
	Cardiac index	↑↑	↓	↓↓
	Stroke volume	↑	↓	↓↓
**Global hemodynamics**	SVR	↓↓	↑↑	↑↑↑
	Mean arterial pressure	↔/slight ↓	↑	↑↑
**LV structure and geometry**	LV end-diastolic diameter	↑ Physiological chamber enlargement	↓	↓↓
	Relative wall thickness	↔	↑	↑↑
	LV mass index	↑ Mild physiological remodeling	↑	↑↑
**Systolic function**	LVEF	↔	↔	↓
	GLS	↔	Impaired	More impaired
**Diastolic function**	e′ velocity	↑	↓	↓↓
	E/e′ ratio	↔	↑	↑↑
	IVRT	↓	↑	↑↑
	Deceleration time	↓	↑	↑↑
**Left atrial remodeling**	LA volume index	↔/slight ↑	↑	↑↑

Abbreviations: LV, left ventricle; GLS, global longitudinal strain; SVR, systemic vascular resistance; LVEF, left ventricular ejection fraction; IVRT, isovolumetric relaxation time; LA, left atrial. Arrows indicate the direction and relative magnitude of change compared with physiological pregnancy.

**Table 2:** Proposed Maternal Cardiovascular Phenotypes in Fetal Growth Restriction and Their Clinical Implications.

	High-Risk Phenotype (Hypodynamic)	Intermediate Phenotype	Low-Risk Phenotype (Near-Normal)
**Clinical**	Early FGR ± preeclampsia	Late FGR	Constitutionally small fetuses
**Maternal cardiovascular adaptation**	Marked maladaptation	Partial maladaptation	Preserved adaptation
**Hemodynamic profile**	↓↓ CO, ↑↑ SVR	↓ CO, ↑ SVR	↔ CO and SVR
**Cardiac remodeling**	Concentric LV remodeling or hypertrophy	Mild	Minimal or absent
**Cardiac function**	Impaired GLS and significant diastolic dysfunction	Mild diastolic dysfunction	Preserved systolic and diastolic function
**Placental disease**	Severe	Moderate	Minimal or absent
**Doppler profile**	Markedly abnormal Doppler	Mild-to-moderate	Normal
**Disease course**	Progressive disease, frequently requiring preterm delivery	Gradual progression	Stable course
**Clinical implications**	Intensive surveillance and structured cardiovascular follow-up	Individualized surveillance	Conservative management
**Therapeutic approach**	Vasodilator	Individualized	Conservative management
**Fourth trimester profile**	Persistent abnormalities	Partial recovery	Near-complete recovery
**Long-term cardiovascular risk**	High	Intermediate	Low

Abbreviations: ↑, increased; ↑↑, markedly increased; ↓, decreased; ↓↓, markedly decreased; ↔, unchanged.

## Data Availability

No new data were created or analyzed in this study. Data sharing is not applicable to this article.
